# Hypercalcemia in a Patient Diagnosed with a Vasoactive Intestinal Peptide Tumor

**DOI:** 10.7759/cureus.6882

**Published:** 2020-02-04

**Authors:** Basma Ataallah, Barjinder S Buttar, Georgia Kulina, Alan Kaell

**Affiliations:** 1 Internal Medicine, Zucker School of Medicine at Mather, New York, USA; 2 Internal Medicine, Northwell Health Mather Hospital, Port Jefferson, USA; 3 Endocrinology, Northwell Health Mather Hospital, Port Jefferson, USA; 4 Medicine, Renaissance School of Medicine, Stony Brook University, Stony Brook, USA

**Keywords:** endocrinology, vasoactive intestinal peptide tumor, vipoma, hypercalcemia

## Abstract

Hypercalcemia is a clinical problem that is commonly seen in both the inpatient and outpatient settings. Overall, most common causes of hypercalcemia include hyperparathyroidism and malignancy. Our case report is the presentation of hypercalcemia in a patient eventually diagnosed with a vasoactive intestinal peptide tumor, a type of neuroendocrine tumor, without associated hyperparathyroidism.

## Introduction

Neuroendocrine tumor (NET) is a rare and heterogeneous tumor type that makes up about 2% of all malignancies​. The neuroendocrine system includes endocrine glands, such as the pituitary, parathyroid, and adrenals, as well as endocrine islet tissue embedded within the thyroid or pancreas ​[1]​. Tumors can develop from any one of these sites.

Vasoactive intestinal peptide tumor (VIPoma) is a type of NET that continuously secretes VIP​. They originate in cells of the gastroenteropancreatic endocrine system and in adrenal or extra-adrenal neurogenic sites​. They are mainly found in the body and tail of the pancreas in 90% of the cases and the tumors tend to be greater than 3 cm in diameter [2]. About 60 to 80% of the VIPomas are malignant and have metastasized at the time of diagnosis. The main metastatic site is the liver, but the lymph nodes, lungs, and kidneys may be involved as well. The classic presentation is watery diarrhea, hypokalemia, and achlorhydria, also known as WDHA syndrome ​[3]​. 

VIPoma are rare tumors with an overall incidence of 0.05% to 2.0%. Only 5% of patients with VIPomas and hypercalcemia have a diagnosis of multiple endocrine neoplasia type 1 (MEN1) syndrome [4]. We present a young female who was admitted with hypercalcemia and found to have metastatic malignant VIPoma without MEN-1 hyperparathyroidism. 

## Case presentation

A 22-year-old female with celiac disease, osteopenia, and depression presented with a two-month history of worsening diffuse abdominal pain, diarrhea, and arthralgia. The patient denied fever, chills, unintentional weight loss, or a history of recent travel. Outpatient labs were significant for a calcium of 15 mg/dL (8.5-10.2 mg/dL), a phosphate of 2.1 mg/dL (2.5-4.5 mg/dL), a potassium of 3.2 meq/L (3.5-5 meq/L), a non-anion gap hyperchloremic metabolic acidosis with chloride of 116 meq/L, a bicarbonate of 18 meq/L, a normal anion gap of 8.5 meq/L corrected for a low albumin of 3 g/dL, and minimally elevated alanine aminotransferase of 61 U/L (7-56 U/L) and aspartate aminotransferase of 55 U/L (10-40 U/L) with normal alkaline phosphatase and bilirubin.

**Figure 1:**
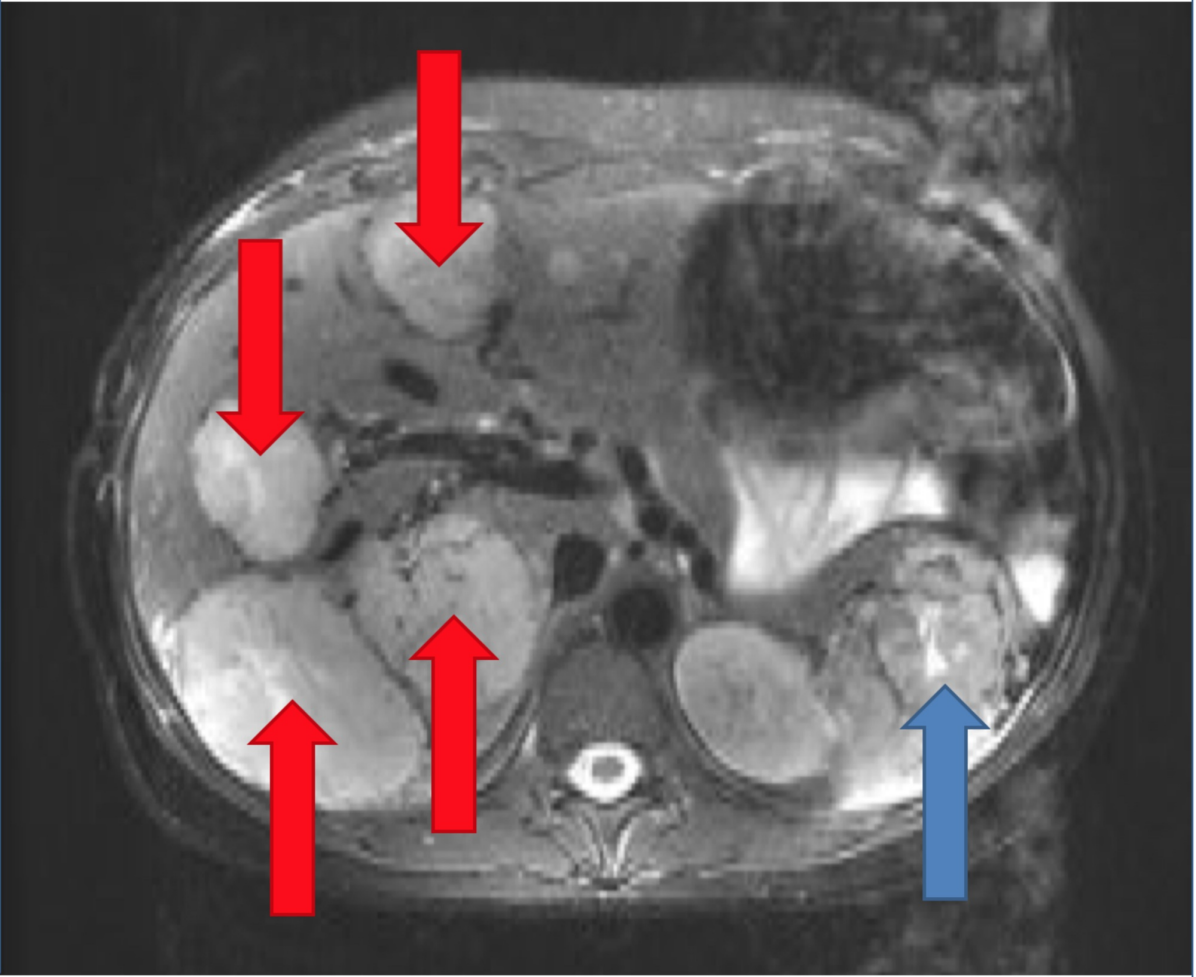
Magnetic Resonance Imaging of the Abdomen Significant for a large, predominantly solid, pancreatic tail lesion with central necrosis (blue arrow). Large liver lesions are present in the majority of the segments of the liver suggesting neuroendocrine metastases (red arrows).

Upon presentation, she was afebrile, and her blood pressure, pulse rate, and respiratory rate were observed to be 120/70 mmHg, 80 beats/minute, and 17 breaths/minute, respectively. Physical examination was notable for mild diffuse abdominal tenderness without signs of rebound. The patient was admitted for evaluation and treatment of severe hypercalcemia. Throughout the hospital course, the calcium improved to 13.9 mg/dL with intravenous normal saline and correction of electrolytes. An abdominal ultrasound revealed two heterogeneous masses in the right hepatic lobe of the liver measuring 7.4 and 8.5 cm. MRI of the abdomen without contrast, followed by MRI abdomen with contrast, confirmed a large, solid, pancreatic tail lesion with liver metastases as shown in Figure 1 [T2].

Additional labs included undetectably low iPTH (intact parathyroid hormone), low PTHrP (parathyroid hormone related peptide), normal renal function, mildly decreased vitamin D of 24 ng/mL, and normal blood glucose level. She was found to have a markedly elevated VIP level of greater than 900 pg/mL (0-60 pg/mL). The diagnosis of hypercalcemia attributed to malignant pancreatic VIPoma with liver metastasis was established. She was given a dose of 4 mg zoledronic acid, which improved her hypercalcemia significantly. She was referred for surgical resection and medical management of her VIPoma with octreotide.

## Discussion

Hypercalcemia can be associated with VIPomas. Of those VIPoma patients with hypercalcemia, up to 5% have treatable hyperparathyroidism associated with MEN-1. The calcium level seen with hyperparathyroidism is typically in the mild range of 10.5 to 11 mg/dL ​[5]​. The majority of VIPomas with hypercalcemia are not attributed to MEN-1 associated hyperparathyroidism. Those cases, as in our patient, have higher calcium levels. Despite the presence of hypercalcemia, patients with VIPoma present with WDHA and not constipation as would be expected in all other cases of hypercalcemia.

Although the pathophysiology of hypercalcemia in VIPoma is unclear, lytic bone lesions do not appear to be a factor even with malignant metastatic VIPomas. The elevated calcium may be multifactorial: dehydration and electrolyte disturbances secondary to diarrhea, secretion by the tumor of a calcitrophic peptide, or bone resorption or PTH-like effects of high VIP levels. [6]

In the setting of VIPoma with hypercalcemia, treatment includes correcting volume and electrolyte abnormalities, calcitonin, and bisphosphonates.

## Conclusions

Our case of a young female, diagnosed first with hypercalcemia, and presenting with the classic WDHA syndrome, was found to have a malignant metastatic VIPoma. Hyperparathyroidism as a part of MEN-1 syndrome was excluded and, therefore, parathyroidectomy not considered. Malignant VIPoma should be considered as a rare but possible underlying cause of patients presenting with similar symptoms.
